# *Pseudomonas taiwanensis* biofilms for continuous conversion of cyclohexanone in drip flow and rotating bed reactors

**DOI:** 10.1002/elsc.202000072

**Published:** 2021-02-02

**Authors:** Ingeborg Heuschkel, Selina Hanisch, Daniel C. Volke, Erik Löfgren, Anna Hoschek, Pablo I. Nikel, Rohan Karande, Katja Bühler

**Affiliations:** ^1^ Department of Solar Materials Helmholtz‐Centre for Environmental Research Leipzig Germany; ^2^ ZINT ‐ Zentrum für integrierte Naturstofftechnik TU Dresden Dresden Germany; ^3^ The Novo Nordisk Foundation Center for Biosustainability Technical University of Denmark Lyngby Denmark; ^4^ SpinChem AB Umeå Sweden

**Keywords:** Baeyer‐Villiger oxidation, biofilm reactors, biotransformation, continuous bioprocess, cyclohexanone monooxygenase

## Abstract

In this study, the biocatalytic performance of a Baeyer‐Villiger monooxygenase (BVMO) catalyzing the reaction of cyclohexanone to ε‐caprolactone was investigated in *Pseudomonas* biofilms. Biofilm growth and development of two *Pseudomonas taiwanensis* VLB120 variants, Ps_BVMO and Ps_BVMO_DGC, were evaluated in drip flow reactors (DFRs) and rotating bed reactors (RBRs). Engineering a hyperactive diguanylate cyclase (DGC) from *Caulobacter crescentus* into Ps_BVMO resulted in faster biofilm growth compared to the control Ps_BVMO strain in the DFRs. The maximum product formation rates of 92 and 87 g m^–2^ d^–1^ were observed for mature Ps_BVMO and Ps_ BVMO_DGC biofilms, respectively. The application of the engineered variants in the RBR was challenged by low biofilm surface coverage (50–60%) of rotating bed cassettes, side‐products formation, oxygen limitation, and a severe drop in production rates with time. By implementing an active oxygen supply mode and a twin capillary spray feed, the biofilm surface coverage was maximized to 70–80%. BVMO activity was severely inhibited by cyclohexanol formation, resulting in a decrease in product formation rates. By controlling the cyclohexanone feed concentration at 4 mM, a stable product formation rate of 14 g m^–2^ d^–1^ and a substrate conversion of 60% was achieved in the RBR.

**Abbreviations**
6OH: 6‐hydroxyhexanoic acid
Aa: adipic acid.
BDW: biofilm dry weight
BVMO: Baeyer‐Villiger monooxygenase
CDW: cell dry weight
CHOL: cyclohexanol
CHON: cyclohexanone
CL: ɛ‐caprolactone
DFR: drip flow reactor
DGC: diguanylate cyclase
RBBR: rotating bed biofilm reactor
RBR: rotating bed reactor

## 1 INTRODUCTION

The Baeyer‐Villiger (BV) oxidation of ketones to the corresponding esters or lactones is an important reaction for the production of pharmaceutical compounds (steroids), fine chemicals (antibiotics, terpenoids), and bulk chemicals (polyester monomers) [1, 2]. Chemically catalyzed BV oxidations often suffer from unstable or explosive chemical oxidants, generate hazardous waste products, and show limited selectivity [3]. Biological BV oxidations, in contrast, performed by flavin‐dependent enzymes, categorized as Baeyer‐Villiger monooxygenases (BVMOs) have attracted considerable attention to overcome these challenges. BVMOs utilize the ‘green’ oxidant oxygen, operate at ambient reaction conditions, generate water as a sole byproduct, and mediate high product selectivity [1, 3, 4]. Yet, industrial applications of BVMOs are restricted due to low enzyme stability and form inhibitory effects of substrate and/or product [3]. The only known industrial example that involves a BVMO catalyzed reaction is the production of a pharmaceutical compound, esomeprazole, on the grams scale (Patent WO2011071982A2).

Extensive studies have been performed to improve BVMO stability by immobilizing enzymes or whole‐cells using artificial techniques, so far with limited success [5, 6, 7]. Application of BVMO‐harboring whole‐cells in a biofilm format comprises a promising bioprocess design. In biofilms, microbial cells immobilize in a self‐produced matrix of extracellular polymeric substances [8, 9]. Robust biofilms sustain microbial growth under harsh process conditions and provide high‐cell‐densities, which could already be exploited to attain high and stable production rates for various oxygenase reactions [10, 11, 12, 13, 14]. In this work, we selected biofilms to investigate the catalytic performance of a BVMO originating from *Acidovorax sp*. CHX 100 [15] for the conversion of cyclohexanone to the corresponding lactone.

**PRACTICAL APPLICATION**

In this work, two alternative biofilm reactor concepts, drip flow reactors (DFRs) and rotating bed reactors (RBRs), were investigated to evaluate biofilm growth and catalytic performance of *Pseudomonas taiwanensis* VLB120 variants. The introduction of an additional diguanylate cyclase (DGC) into *Pseudomonas taiwanensis* VLB120 biofilms resulted in faster biofilm formation than the control strain within DFRs. For RBR, an active oxygen supply mode combined with a twin capillary spray feed strategy resulted in 70–80% of the biofilm surface coverage. However, the Baeyer‐Villiger monooxygenase (BVMO) was severely inhibited by the byproduct cyclohexanol, resulting in decreased production rates. By controlling the cyclohexanone feed, a stable product formation and a substrate conversion of 60% was achieved in the RBR. Overall, this work highlights the possibility of establishing biofilms in RBRs to accomplish continuous biocatalytic synthesis.

The choice of a biofilm reactor type as well as operating conditions highly influences the overall performance of biofilms [16]. Different types of continuous flow biofilm reactors, including drip‐flow, miniaturized flow, packed bed, trickle bed, and rotating bed reactors, are known [17, 18, 19, 20]. These reactor formats show considerable differences in the specific available surface area, oxygen transfer capacities, and the mechanisms employed to remove excess biomass [16].

In drip‐flow reactors (DFRs), the medium drips onto a carrier surface and consequently, flows across it in a plug‐flow manner [21]. Such systems are ideal for general biofilm studies, such as screening specific mutants or growth materials for biofilm formation [19, 22]. In DFRs, the biofilm is grown under low fluidic shear and close to the air‐liquid interface for attaining a high oxygen mass transfer [21, 22]. These operational conditions result in a thick and heterogeneous biofilm. For technical applications, a biofilm reactor system that stimulates a moderate to high‐shear environment to establish a thin, dense, and homogenous biofilm is beneficial. Rotating bed biofilm reactors (RBBRs) is advantageous, as the rotational speed of discs or coupons can generate high shear rates necessary to attain biofilm growth and structure suitable for biocatalytic purposes [22]. We aimed to investigate both biofilm reactors, DFRs and RBBRs, to study the catalytic performance of recombinant *Pseudomonas taiwanensis* VLB120 biofilms for the production of polycaprolactone monomers. Polycaprolactone is a biodegradable [23] and highly versatile polymer produced annually at a multi‐thousand scale [24]. Its mechanical properties, and miscibility with a large range of other polymers, are interesting in the production of scaffolds for tissue engineering [25, 26], long‐term drug delivery [27], microelectronics [28] and packaging [27].

Two *P. taiwanensis* VLB120 variants, *P. taiwanensis* VLB120_BVMO (hereafter Ps_BVMO), and *P. taiwanensis* VLB120_BVMO_DGC (hereafter Ps_BVMO_DGC), were studied regarding biofilm formation and biocatalytic activity in DFRs and RBBRs. Here, Ps_BVMO_DGC, with an additional plasmid harboring a diguanylate cyclase, facilitated faster biofilm formation compared to the Ps_BVMO strain. A maximum production rate of 92 g m^–2^ d^–1^ and a cyclohexanone conversion of 74% was obtained after 6 days of biofilm cultivation in the DFR. In RBBR, gas‐liquid interfacial forces (imposed by the centrifugal force) resulted in a dense and stable biofilm. Improvement of oxygen availability, medium and substrate feed in the RBBR, resulted in a stable production rate of 14 g m^–2^ d^–1^ with 60% cyclohexanone conversion for 169 h.

## 2 MATERIALS AND METHODS

### 2.1 Chemicals

Chemicals for growth medium and HPLC mobile phase were purchased in the highest purity available from Carl‐Roth GmbH (Karlsruhe, Germany), Merck (Darmstadt, Germany), or Sigma‐Aldrich (Steinheim, Germany). Cyclohexanone and cyclohexanol were purchased with a purity of ≥99.5% purity from Sigma‐Aldrich (Steinheim, Germany). ε‐caprolactone and adipic acid were obtained at 99% purity from Alfa Aeser (Kandel, Germany) and AppliChem GmbH (Darmstadt, Germany), respectively. 6‐Hydroxyhexanoic acid was purchased from abcr GmbH (Karlsruhe, Germany), purity 95%, and used without further purification.

### 2.2 Bacterial strains and plasmids

Bacterial strains and plasmids used for cloning procedures are provided in the supporting information (Table S1 and S2). *Pseudomonas taiwanensis* VLB120 was transformed with the plasmid pRSF_Ptrc1O::BVMO, harboring a Baeyer‐Villiger monooxygenase (BVMO) from *Acidovorax* sp. strain CHX100, resulting in the recombinant strain *P. taiwanensis* VLB120 pRSF_Ptrc1O:BVMO (Ps_BVMO). The expression system was based on the broad‐host‐range vector pPMQAK1 (RSF ori), and BVMO production was posed under the control of the P_trc1O_ promoter [29]. Furthermore, the Ps_BVMO strain was transformed with the plasmid pS6311::DGC‐244 containing a diguanylate cyclase (see below), resulting in *P. taiwanensis* VLB120/pRSF_Ptrc1O:BVMO/pS6311::DGC‐244 (Ps_BVMO_DGC). A mutant variant of DGC from *Caulobacter crescentus*, termed DGC‐244 [30], was used to construct plasmids for inducible biofilm formation and was a kind gift from Prof. Urs Jenal (Biozentrum, University of Basel). This mutant lacks feedback inhibition by cyclic diguanylate.

### 2.3 Cultivation of *Pseudomonas taiwanensis* VLB120 strains

All cultivations were carried out in Multitron Pro shaker (Infors, Bottmingen, Switzerland) at 30°C and 200 rpm (2.5 cm amplitude). Overnight cultures (10 mL Lennox broth) [31] were inoculated directly form a 10% (v/v) glycerol cryo‐stock and used for inoculation (1% v/v) of 100 mL M9* medium [32]. Cultures were incubated for 12–16 h in 1‐L baffled shake flasks. When appropriate, Kanamycin and Gentamycin were added to the culture medium as selection markers at concentrations of 50 and 10 μg mL^–1^, respectively.

### 2.4 Technical setting of the reactor systems

#### 2.4.1 Biofilm cultivation in the drip flow reactor setup

Nylon coupons were mounted at the bottom of custom‐made glass cylinders (inner diameter = 10 mm, height = 100 mm) at a 45° angle. M9* minimal growth medium (supplemented with 28 mM glucose) was continuously supplied at a flow rate of 100 μL min^–1^ (in a drip‐flow manner) and then removed via Tygon tubing (LMT‐55, inner diameter = 2.06 mm, 0.88 mm wall thickness, Ismatec, Wertheim, Germany) using a peristaltic multichannel pump (type 530S, Watson‐Marlow Fluid Technology Group, Falmouth, UK) containing Marprene pump tubing (MHLL, inner diameter = 2.06 mm, 0.88 mm wall thickness, Watson‐Marlow Fluid Technology Group, Falmouth, UK). Cultivation was performed at room temperature (RT, 22–26°C). During biotransformation, the flow rate was decreased to 52 μL min^–1^.

#### 2.4.2 Biofilm cultivation in the RBBR

**FIGURE 1:**
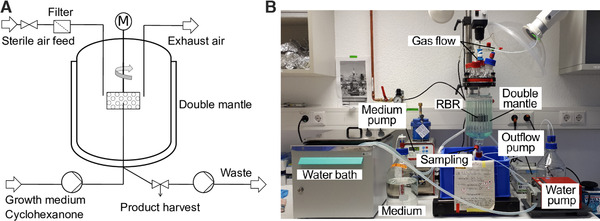
Schematic representation of a rotating bed reactor (A), and image of the laboratory setup (B). For further details, please see the text

The RBR reactor model S311 was provided by SpinChem® (Umeå, Sweden), which operates with a metal compartment, including four custom‐made 3D printed Nylon inserts fabricated by SpinChem consisting of 17 lamellas each (height 2 mm) (Figure 1). M9* medium (supplemented with 28 mM glucose) was continuously pumped through PTFE tubing from the bottom of the vessel (piston pump MCP‐CPF Process IP65, Ismatec, Wertheim, Germany). It was sparged to the inner part of the metal compartment by using one or two custom made metal capillaries, respectively (i.d. 2 mm and 0.6 mm for single and double capillaries). The waste medium was removed by a peristaltic multichannel pump (IPC, Ismatec, Wertheim, Germany). Cultivation was performed at 30°C. Oxygen transfer was initiated either in a passive manner by using a sterile filter at the top of the vessel or in an active mode by using a pressurized airflow at 100 mL min^–1^ (InFlux flow meter, Alresford, UK). Gas‐phase samples were collected in a custom‐made bubble trap at the gas outlet, and liquid samples were taken at the reactor bottom through one of the two available sampling ports.

### 2.5 Inoculation of the reactor system

The outlets of DFRs and RBBRs were closed and the vessel filled with 20 and 200 mL M9* pre‐culture (OD_450_ = 5) for inoculation, respectively. The cartridge of the RBBR was rotated at 200 rpm, while the coupon in the drip flow reactors was kept submerged for 2 h to facilitate cell attachment at the substratum surface. After 2 h, the cell suspension was removed from the vessels, and the medium flow was initiated.

### 2.6 Harvest and biomass determination

In the case of DFRs, biomass was carefully removed from the coupon with a spatula. In the RBBRs, biomass from the lamella was removed with pressurized air. Subsequently, the surfaces were rinsed with medium to remove residual biofilm chunks. Collected biomass was centrifuged (3000 rpm, 10 min, 4°C), and supernatants were removed by pipetting and drying at 80°C in an oven (Model 56, Binder GmbH, Tuttlingen) for one week. Biomass amounts were determined by weighing the dried samples.

### 2.7 Oxygen quantification by gas chromatography

For oxygen quantification, a Trace 1310 gas chromatograph (Thermo Fisher Scientific, Waltham, MA) equipped with a TG‐BOND Msieve 5A capillary column (30 m, I.D.: 0.32 mm, film thickness: 30 μm, ThermoFisher Scientific) and a thermal conductivity detector operating at 100°C with a filament temperature of 300°C and a reference gas flow rate of 2 mL min^−1^ was applied. Argon gas was utilized as carrier gas at a constant flow rate of 5 mL min^−1^. Samples were manually injected with airtight Hamilton syringes (Hamilton, Reno, USA) at an injection temperature of 50°C and a split ratio of 2. For separation, the oven temperature was kept constant at 35°C for 3 min.

### 2.8 Quantification of cyclohexanone, cyclohexanol, and ε‐caprolactone by gas chromatography (GC)

Reactants were extracted from samples (1 mL) collected from the outlet with ice‐cold diethyl ether (500 μL, containing 0.2 mM decane as internal standard) by vigorous mixing for 2 min followed by centrifugation (17,000 g, 2 min, 4°C). The ether phase was separated and dried over anhydrous Na_2_SO_4_. Cyclohexanol, cyclohexanone, and ε‐caprolactone were quantified using a GC Trace 1310 (Thermo Fisher Scientific) equipped with a TG‐5MS capillary column (5% diphenyl/95% dimethyl polysiloxane, 30 m, i.d., 0.25 mm, film thickness: 0.25 μm, Thermo Fisher Scientific) equipped with a TG‐5MS capillary column (5% diphenyl/95% dimethyl polysiloxane, 30 m, I.D., 0.25 mm, film thickness: 0.25 μm, Thermo Fisher Scientific) and a flame ionization detector operated at 320°C, 350 mL min^−1^ air, 30 mL min^−1^ makeup gas (N_2_), and 35 mL min^−1^ hydrogen gas flow rates. N_2_ was applied as carrier gas at a constant flow of 1.5 mL min^−1^. An injection volume of 1 μL sample was applied onto the column using a PTV injector (40°C), programmed with a temperature gradient of 2°C s^−1^ from 40 to 250°C (1 min hold at 250°C), followed by a cleaning phase of 5 min at 450°C. A split ratio of 7 was applied (split‐flow 11 mL min^–1^). The oven temperature profile was: 40°C for 1 min, 40–80°C at 10°C min^−1^, 80–320°C at 100°C min^−1^, and 320°C for 3 min.

### 2.9 Quantification of ε‐caprolactone, 6‐hydroxyhexanoic acid, and adipic acid using high‐performance liquid chromatography (HPLC)

Samples (500 μL) were collected from the outlet, centrifuged (17000 g, 7 min, 4°C) and utilized for HPLC analysis (Dionex Ultimate 300, Thermo Fisher Scientific). To detect ε‐caprolactone, 6‐hydroxyhexanoic acid, and adipic acid, the samples were acidified with 1 M HCL before HPLC analysis with a ratio of 1:5. A Dionex Ultimate 300, Thermo Fisher Scientific separation module equipped with a variable wavelength detector operating at 210 nm was used. An injection volume of 20 μL onto an Acclaim organic acid column (150 cm length, 3.0 mm diameter, 3 μm particle size, ThermoFisher Scientific) was applied. The mobile phase consisted of MilliQ water (A), 100 mM Na_2_SO_4_ (B) (pH 3, adjusted with methanesulfonic acid), and pure acetonitrile (C). The column oven was kept constant at a temperature of 60°C. The column was equilibrated for 2 min at the initial eluent composition of 95% B and 5% C. A binary elution gradient with 0.4 mL min^–1^ over 7 min was applied from 95 to 20% mobile phase B with subsequent regeneration of the column with 20% B for 1 min and washing the column with a 95% mobile phase B for 2 min.

## 3 RESULTS

*P. taiwanensis* VLB120 was selected as a host organism because of its biofilm‐forming capacity and resilience towards toxic compounds [33, 34, 35]. A BVMO derived from *Acidovorax* sp. CHX100 shows an exceptionally high whole‐cell specific activity of 160–180 U g_CDW_^–1^ [36, 37] towards cyclohexanone oxidation and was thus genetically introduced into the *Pseudomonas* strain as the model reaction system (Ps_BVMO). In addition, a plasmid harboring a diguanylate cyclase (DGC), which catalyzes the reaction of two guanosine‐5′‐triphosphate (GTP) molecules to form c‐di‐GMP, was introduced (Ps_BVMO_DGC). C‐di‐GMP is a second messenger molecule, involved in the molecular switch between planktonic and biofilm lifestyles [38, 39, 40], and its presence usually facilitates biofilm formation. The difference in Ps_BVMO and Ps_BVMO_DGC biofilm growth, development, and catalytic performance were evaluated in the DFR and RBR setups.

### 3.1 Presence of diguanylate cyclase improves biofilm growth in the DFR setup

**FIGURE 2:**
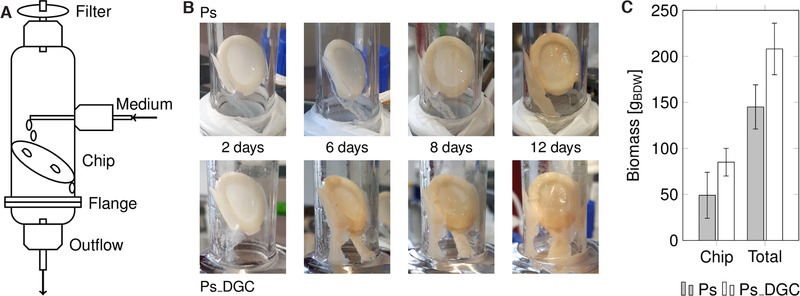
Evaluation of biofilm growth on the nylon coupon surface in the DFR. (A) Schematic representation of the DFR setup. (B) Pictures of Ps_BVMO (Ps) and Ps_BVMO_DGC (Ps_DGC) biofilm development with time. (C) The amount of biomass generated after 12 days of biofilm cultivation in the drip‐flow reactor. Error bars depict the standard deviation of two individual biological replicates

For the DFR, a 3D printed nylon coupon was mounted inside a glass cylinder at a 45° angle (Figure 2A, Figure S2). A sterile filter on top of the cylinder ensured a passive mode of oxygen supply by exchanging the air phase. After inoculation, both strains settled on the nylon coupons and the reactor's inner glass surface. The mild shear forces exhibited by the drip‐flow resulted in a thick and inhomogeneous distribution of the biofilm on the coupon and glass surface (Figure 2B, Figure S3). After 12 days of cultivation, biofilm formation on the coupons differed significantly between the two strains (208 mg_BDW_ vs. 145 mg_BDW_ for Ps_BVMO_DGC and Ps_BVMO, respectively). As already observed before, the diguanylate cyclase expression leads to fast and high biofilm formation [41]. Pellicle formation on the air‐liquid interface in the efflux bottle was observed only for Ps_BVMO_DGC (Figure S1).

### 3.2 Biocatalytic performance of *Pseudomonas* variants' varied between the early induced biofilms and late induced mature biofilms

**FIGURE 3:**
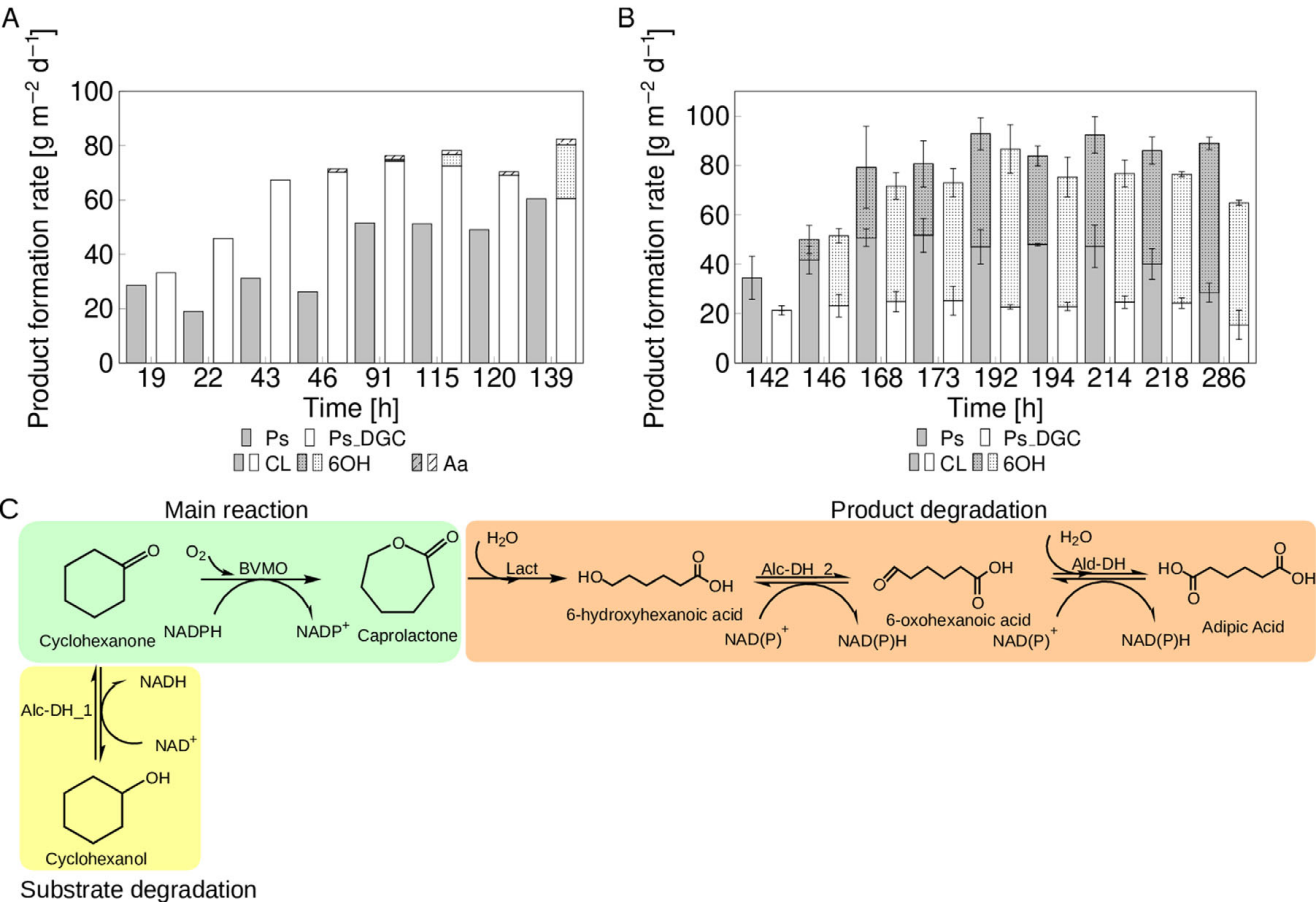
Catalytic performance of Ps_BVMO (Ps) and Ps_BVMO_DGC (Ps_DGC) biofilms over time in DFRs. (A) The biotransformation was initiated directly after the inoculation step. Sampling started 19 h after inoculation. (B) The biotransformation was initiated after 5 days (120 h) of biofilm growth; 142 h after inoculation, product determination was started. The production rates were calculated based on the surface area of the coupon (3.09 cm^2^). The main product caprolactone (CL), and three side‐products were analyzed: ε‐6‐hydroxyhexanoic acid (6OH), and adipic acid (Aa) and cyclohexanol (CHOL). The CHOL concentration was below 0.15 mM in all cases. Error bars depict the standard deviation of two individual biological replicates. (C) Schematic representation of the main reactions, substrate and product degradation. Alc‐DH_1, alcohol dehydrogenase 1; Alc‐DH_2, alcohol dehydrogenase 2; Ald‐DH, aldehyde dehydrogenase; BVMO, Baeyer‐Villiger monooxygenase; Lact, lactonase

We aimed to investigate if high biomass presence translates into high catalytic performance in the DFR. The two *Pseudomonas* variants grown in the DFR were analyzed in two different conditions. In the first set of DFR experiments, the biotransformation reaction was initiated directly after inoculating the DFR, and for the second set of experiments after 5 days. The biofilm growth rate and its catalytic performance for both variants were monitored. When the biotransformation was initiated directly after inoculation, the Ps_BVMO_DGC biofilm showed higher product formation rates as compared to the variant without the additional cyclase. Average production rates of 76 and 51 g m^–2^ d^–1^ (Figure 3A, samples 91–139 h) were obtained for Ps_BVMO_DGC and Ps_BVMO biofilms, respectively. During the start phase of the biotransformation (first 43 h) both variants exclusively produced ɛ‐caprolactone (CL) as the main product. In the later phase of biotransformation, CL degradation to 6‐hydroxyhexanoic acid (6OH), most likely by strain‐inherent lactonases, was observed for Ps_BVMO_DGC. Also, 6OH was further degraded to adipic acid (Aa), probably due to host intrinsic dehydrogenases. In addition, the substrate (cyclohexanone) was converted to cyclohexanol (CHOL), most likely by strain‐inherent dehydrogenases. The observed substrate and product degradation are depicted in Figure 3C. Cyclohexanol is known to severely inhibit the here applied BVMO at high concentrations [37]. In the current experiments, CHOL levels were low and stayed below 0.1 mM during the entire biotransformation. Overall, the production rate was 1.5‐fold higher for Ps_BVMO_DGC variant compared to the Ps_BVMO variant when the inoculation was performed simultaneously at the start of the biotransformation.

In the second set of DFR experiments, the biotransformation reaction was initiated 120 h after inoculation to explore the reaction performance of well‐grown (mature) Ps_BVMO_DGC and Ps_BVMO biofilms (Figure 3B). In this case, gene expression was not induced right from the start, resulting in higher biomass concentrations (157 mg m^–2^ Ps_BVMO and 274 mg m^–2^ Ps_ BVMO_DGC). Within 24 h of adaptation to the biotransformation conditions, both strains reached a maximal product formation rate of 92 and 87 g m^–2^ d^–1^ for Ps_BVMO and Ps_ BVMO_DGC, respectively. In comparison to early‐stage biofilms, the mature biofilm resulted in the formation of 6OH for both strains.

Overall, the Pseudomonas variants' biocatalytic performance varied between the early induced biofilms and late induced mature biofilms. For early‐stage biofilms, Ps_BVMO showed lower biocatalytic rates, which might be attributed to the lower biomass production (13 g m^–2^) compared to the Ps_ BVMO_DGC variant (227 g m^–2^). Here, CL was the major product formed for both variants with a minor amount of side‐products (Figure 3A). However, the surplus in biomass is not reflected by the productivity of the system, indicating a considerable amount of EPS or otherwise unproductive biological matter. For the late induced biofilms, both variants showed almost similar catalytic rates with comparable biofilm growth. Striking is the significant increase of the side product (6OH), which was detected in both variants, likewise in mature biofilms. Perhaps the supply of cyclohexanone to non‐adapted cells resulted in enhanced cell lysis and a release of lactonases into the EPS. Nevertheless, more experiments are necessary to clarify this phenomenon.

In conclusion, the DFRs experiments showed high product formation rates (above 87 g m^–2^ d^–1^), which might result from the excellent oxygen mass transfer attributing from the high air‐liquid interfacial area. However, the biofilm structure and distribution were inhomogeneous, mainly due to the low‐shear environment. In this context, the application of a rotating bed biofilm reactor (RBBR) format to impose moderate shear stress by using rotational speed to establish thin and homogenous biofilm growth was investigated next.

### 3.3 BVMO stability a key issue for continuous cyclohexanone oxidation in the RBBR

The SpinChem® rotating bed reactor (RBR) Model S3 was used to study biofilm growth and catalytic performance on the RBBR cassette under shear stress imposed by the rotational speed. Further modifications of the RBBR operation were accomplished to mimic the high air‐liquid interface attained in the drip‐flow system. These modifications include localizing the rotating bed reactor in the air phase and supplying the medium via a nozzle in a “spray feed fashion” in a radial direction. Spraying was performed intermittently at a flow rate of 0.42 mL min^–1^ as 1‐second pulses followed by 20‐s pauses. During biofilm cultivation, the cartridge was rotated at 200 rpm, and the medium droplets were passed through the 3D‐printed plastic inlets driven by centrifugal force. Oxygen consumed within the system was exchanged in a passive mode by using a sterile filter at the top of the reactor lid.

**FIGURE 4:**
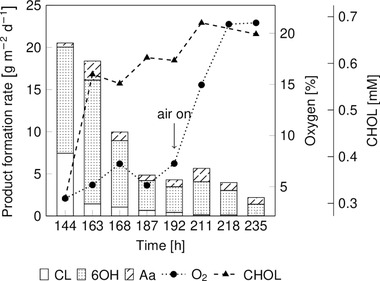
The catalytic performance of Ps_BVMO biofilm over time in RBBR. Ps_BVMO was grown for 5 days (120 h) at 200 rpm, prior to the biotransformation. M9* medium (28 mM glucose) was supplied at a flow rate of 0.42 mL min^–1^ with 1 s pulses of medium followed by 20 s pause. The addition of 1 mM IPTG initiated BVMO synthesis, and 4 mM cyclohexanone was provided via the growth medium. Sampling was started 144 h after inoculation. After 192 h, the air feed was established with a flow rate of 100 mL min^–1^ using pressurized air

For the RBBR experiment shown in Figure 4, the Ps_BVMO strain was selected because it reached a higher production rate in (late induced) mature‐stage biofilms compared to Ps_BVMO_DGC in the DFRs (Figure 3B). Similar to the drip‐flow setup, biotransformation was initiated five days after inoculation. On the sixth day (144 h), 70% of 4 mM cyclohexanone was converted, corresponding to a production rate of 20 g m^–2^ d^–1^. Concomitantly, the oxygen content within the reactor system dropped below 10% (v/v) of total oxygen in the gas phase. The CL production rate dropped over time, while the CHOL concentration increased rapidly from 0.3 to 0.6 mM, indicating that CHOL might inhibit BVMO catalytic activity. Although a pressurized air feed was installed on day 8 to improve the oxygen supply in the reactor system, the ε‐caprolactone production rate did not recover despite an increase in the oxygen concentration of the air phase.

After 235 h, biofilm growth and biotransformation were terminated, and the biomass was removed from the nylon inserts. The surface coverage of the RBBR cartridge with biofilm was about 50–60%, accounting for a total biomass amount of 320 mg_BDW,_ which corresponds to 29.34 g m^–2^. Although the overall product pattern was comparable to the cultivation in the DFR, the maximum production rate was about fourfold lower, which is in line with lower biomass presence. Further modifications in the RBBR system and reaction conditions are necessary to facilitate faster biofilm growth, higher biofilm surface coverage, and stable production rates. In this context, Ps_BVMO_DGC was selected for all further experiments.

### 3.4 The selection of Ps_BVMO_DGC strain with a modified medium spray feed improved biofilm surface coverage but not production rates

Based on the RBBR results obtained above, the feed line was modified. Here, a second nozzle was introduced into the system to ensure an equal distribution of medium throughout the cultivation module. Furthermore, the cyclase containing variant Ps_BVMO_DGC was chosen as the biocatalyst. Modifying the feed line resulted in significantly enhanced biofilm coverage of the cultivation module (up to approx. 80%) and was therefore applied for all subsequent cultivations.

**FIGURE 5:**
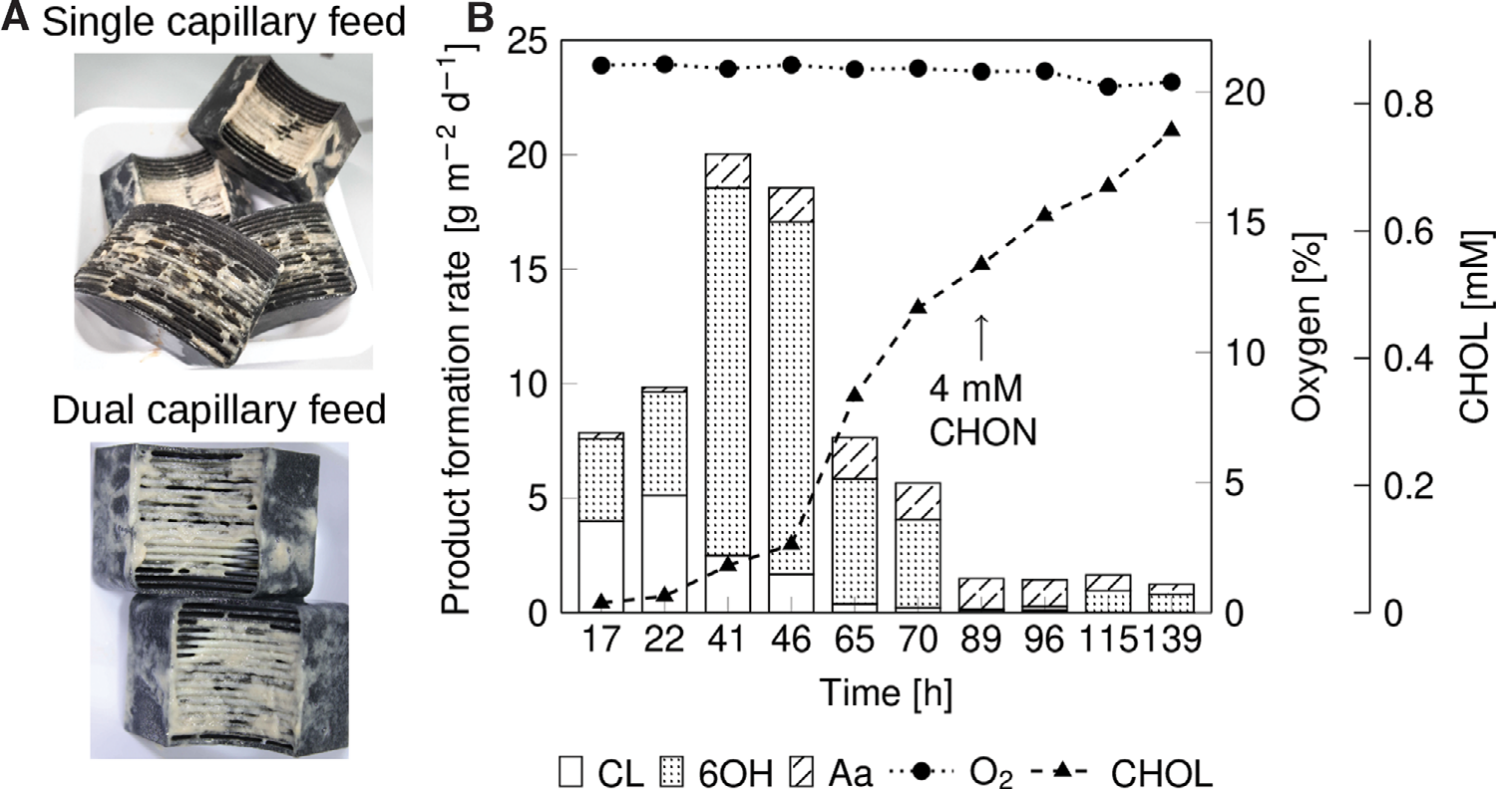
(A) Change in the biofilm surface coverage using Ps_BVMO_DGC based on the feeding strategy. (B) Catalytic performance of Ps_BVMO_DGC biofilm based on dual capillary medium spray feed. The flow rate of M9* (14 mM glucose, 1 mM IPTG) medium was increased to 0.64 mL min^‐1^ (1 s spray, 20 s pause), and the RBBR rotated at 400 rpm. CHON (7 mM) was added at the beginning of the cultivation, and the feed concentration was reduced to 4 mM on the third day. A continuous air feed of 100 mL min^‐1^ was supplied to provide fresh oxygen for growth and reaction

The RBBR reactor was inoculated with Ps_BVMO_DGC, and simultaneously, the biotransformation was initiated by the addition of 7 mM cyclohexanone (Figure 5B). The oxygen concentration in the reactor was stable at the saturation concentration of 21% for the entire experiment due to the continuous feed of pressurized air. The production rate increased to a maximum of 20 g m^–2^ d^–1^ within the first 41 h, followed by a drop to 7.7 g m^–2^ d^–1^, similar to our previous observation (Figures 5B and  4). Concomitantly, a steep increase in the CHOL amount from 0.1 to 0.34 mM, was observed again. Therefore, the CHON feed concentration was reduced to 4 mM after 89 h to reduce CHOL formation. However, the overall biofilm activity dropped further below 1.5 g m^–2^ d^–1^, while the CHOL production increased to 0.7 mM with small amounts of Aa. This points to strong BVMO inhibition by CHOL.

Overall, the biofilm surface coverage on the RBBR cartridge was improved to 80%, and biofilm ‘spikes’ were also observed during reactor operation (Figure S5). However, improvement in surface coverage could not be transferred to biofilm productivity. To establish stable biofilm activity for CHON oxidation, an optimal substrate concentration that allows minimal byproduct formation and high CL production needs to be identified.

### 3.5 Optimizing cyclohexanone feeding to stabilize biofilm activity

**FIGURE 6:**
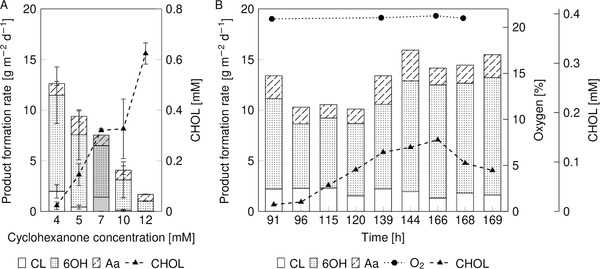
(A) The impact of the cyclohexanone feed on the product formation rate and cyclohexanol formation. Error bars represent the standard deviation for three individual samples collected during 2–3 days of biotransformation. The dark grey bar depicts the averaged product formation rates of a 6‐day reactor run with a feed concentration of 7 mM (Figure 5). (B) A cyclohexanone feed concentration of 4 mM was chosen to examine the stability of the biofilm activity in RBBR. The rotation speed was increased from 200 to 400 rpm after 144 h to evaluate the impact on the catalytic performance

It was assumed that the high CHOL formation rates strongly inhibited the BVMO activity. An optimal substrate concentration that allows minimal byproduct formation and high CL production needs to be identified to establish stable biofilm activity for CHON oxidation. Therefore, the CHON feed concentration was varied from 4 to 12 mM in the inlet medium flow. A starting CHON concentration of 4 mM was chosen because the apparent *K*_S_ value for CHON was 1.9 mM for Ps_BVMO biofilm (data not shown here). With increasing cyclohexanone feed concentrations, the product formation rate, mainly 6OH, dropped from 12.5 to only 1.5 g m^–2^ d^–1^. This decrease in production rate might be due to inhibitory effects caused by the rising CHOL concentration (Figure 6A). The lowest cyclohexanone feed concentration of 4 mM was chosen to examine the long‐term stability of the biofilm activity. In a freshly inoculated RBBR the CHON feed concentration was reduced to 4 mM (Figure 6B). Thereby, stable biotransformation with a maximum product formation rate of 16 g m^–2^ d^–1^ could be achieved for at least 169 h before terminating the experiment. The primary biotransformation product was detected to be 6OH with 15% CL and 13% Aa, which corresponds to an overall CHON conversion of 60%. In this case, the CHOL concentration remained below 0.17 mM, further pointing to the CHOL‐sensitivity of the BVMO.

## 4 DISCUSSION

### 4.1 Expression of *dgc* encoding a feedback‐resistant diguanylate cyclase increases ab initio productivities due to faster biofilm formation

*Pseudomonas taiwanensis* VLB120 is considered to be a good biofilm former but necessitates 5–7 days of cultivation time to form a mature biofilm [42, 43]. To reduce biofilm formation time, an additional DGC was introduced into *Pseudomonas*. DGCs catalyze two molecules of GTP to form c‐di‐GMP, a global messenger molecule involved in the switch between planktonic and biofilm lifestyles [38, 39, 40]. A beneficial effect of increasing c‐di‐GMP levels on catalytic biofilm performance was reported for *Pseudomonas putida* KT2440 in the context of dehalogenation activity [44]. Here, we evaluated the impact of DGC activity on *Pseudomonas* biofilm growth and catalytic performance. For the catalytic performance studied during the initial biofilm growth phase, the strain harboring the DGC produced approximately 1.5‐fold more product than the control strain (Figure 3A). A similar observation was reported by Schmutzler and coworkers, for the epoxidation of styrene [40]. However, the biomass production was 17‐fold higher in the DGC strain compared to the control strain. When the biofilm was cultivated for 6 days before initiating the biotransformation, the Ps_BVMO biofilm resulted in slightly higher product formation than the Ps_BVMO_DGC biofilm. Here, Ps_BVMO_DGC also produced (1.4 fold) higher biomass, but the total production rate was reduced by 10–20%, which might indicate mass transfer limitations (Figure 3B).

Initiation of reaction simultaneously to inoculation, the maximal productivity was reached using Ps_BVMO_DGC biofilm after 4 days of cultivation (Figure 3A). Accordingly, the DGC variant was able to decrease the biofilm cultivation time by 50%. Nevertheless, controlling biofilm thickness is necessary to overcome excess biomass and, subsequently, mass transfer limitations. Whereas biofilms are considered to be the robust whole‐cell catalytic format, their applications on the technical scale are challenged by a substrate and/or product degradation, as observed in this work.

### 4.2 Overcoming cyclohexanol inhibition of BVMO to stabilize biofilm activity

The continuous operation of cyclohexanone oxidation in the RBBR was restricted due to the drop in Ps_BVMO_DGC biofilm activity (Figure 5B). This loss in the biofilm activity might come from the toxic or inhibition effects of the substrate, products, or byproducts. Such effects were already observed in other biofilm studies. These include *Pseudomonas* biofilms consisting of a cytochrome P450 monooxygenase catalyzing cyclohexane oxidation [35] or a styrene monooxygenase catalyzing styrene to the corresponding epoxide [34].

A severe impact of cyclohexanol inhibition on BVMOs has already been described in the literature [45, 46, 47, 48]. Mallin and coworkers investigated the effect of CHOL on an isolated BVMO from *A. calcoaceticus*, and reported a 70% drop in the initial activity in the presence of 10 mM CHOL [45]. To overcome substrate inhibition, Mallin and coworkers encapsulated *E. coli* cells harboring a BVMO from *Acinetobacter calcoaceticus* NCIMB 9871 in alginate beads and investigated reaction performance in an RBBR at 20 mM cyclohexanone concentration. Here, a CHON conversion of 36% ± 6.1% (20 mM, 500 rpm) was achieved in 24 h [45].

For the BVMO used in the present study, inhibition of the isolated enzyme was observed already at 0.1–0.2 mM CHOL (data not shown here). Also, resting‐cell activities of Ps_BVMO cells dropped by 50% upon the presence of 0.4–0.5 mM CHOL [37]. To minimize the inhibitory effects of substrate and product on the BVMO, Hilker and coworkers demonstrated *an in situ* substrate feeding and product removal strategy using an adsorbent resin [49]. This strategy was successfully demonstrated for recombinant *E. coli* expressing BVMO on the preparative scale, where 25 g of lactone was produced with a 75–80% product yield. An alternative option is to screen BVMOs that are not inhibited by CHOL and apply them for process development.

### 4.3 Product degradation as a key challenge for continuous caprolactone production

In the present work, product degradation to 6OH was mainly observed in the mature biofilms of both variants in DFRs (Figure 3A,B). However, the change in the reactor format to RBBR resulted in product degradation by the early‐stage biofilm and mature biofilm (Figure 4). Besides, 6OH was further degraded to Aa by host intrinsic dehydrogenases. A ring‐opening of CL to 6OH might come from the host intrinsic hydrolase enzyme either present intracellular or extracellular within the biofilm EPS. It has been demonstrated that the change in the operating conditions and reactor formats could significantly affect biofilm architecture and could also alter the EPS composition [50, 51]. In our case, such changes might trigger CL degradation, but further investigation on biofilm proteome at different growth stages is necessary to validate this assumption. Overall, strategies to overcome substrate and product degradation need to be established to maximize CL yield. This includes knocking out the (dehydrogenases and/or hydrolase) genes responsible for the degradation of substrate and product. Another strategy would be to screen (whole‐cell) biocatalysts with lower substrate and product degradation rates than the recombinant *P. taiwanensis* VLB120 strain.

### 4.4 Optimization of RBBR system to improve biofilm development

The biofilm product formation rates for polycaprolactone monomers in the DFR systems were 4–5 fold higher than in the RBBR systems. One option to optimize the RBBR system is to change the medium feeding strategy for maximizing the biofilm surface coverage to 100%. In this context, changing from non‐submerged to submerged mode might be beneficial. Furthermore, the rotation speed of the RBBR may impact biofilm development towards a thin and dense film and needs to be investigated more closely. Finally, recycling of the medium needs to be ensured for efficient resource utilization due to the incomplete substrate conversion of 60–70%. The RBBRs offer a promising scale‐up concept by stacking several rotating bed compartments on top of each other, thereby increasing the available surface area for biofilm growth. Different biofilm‐based catalysis can now be exploited to investigate the RBBR performance compared to other biofilm reactors.

## 5 CONCLUSION

In this work, two alternative reactor concepts, DFRs and RBBRs, were evaluated and developed to perform biofilm‐based continuous catalysis by using *Pseudomonas* variants. The introduction of an additional diguanylate cyclase (DGC) into Ps_BVMO, resulted in faster biofilm formation with higher cyclohexanone oxidation rates of up to 76 g m^–2^ d^–1^ compared to 51 g m^–2^ d^–1^ in the control strain. However, enhanced biofilm growth might lead to mass transfer issues resulting in the cohort of inactive biomass. The BVMO used in this study is highly sensitive to CHOL. This byproduct inhibition was the main reason for the drop in biofilm activity. By reducing the CHON inlet concentration to 4 mM, it was possible to maintain a low level of CHOL and achieve a stable product formation rate of 14 g m^–2^ d^–1^ at a substrate conversion of 60%. Product degradation to multiple side products was observed in both reactor setups, and future research efforts are necessary to minimize degradation rates. Overall, this work now opens up the possibility of establishing biofilms in RBRs to accomplish continuous biocatalytic synthesis.

## CONFLICT OF INTEREST

The authors have declared no conflict of interest.

## Supporting information



Supplementary information

## Data Availability

The data that support the findings of this study are available from the corresponding author upon reasonable request.
