# Supplementary material for: Pseudomonas taiwanensis biofilms for continuous conversion of cyclohexanone in drip flow and rotating bed reactors
Source: Eng Life Sci. 2021 Feb 2;21(3-4):258–69. doi: 10.1002/elsc.202000072 (PMC7923564; doi:10.1002/elsc.202000072)
Supplement: Supplementary file 1 — Supplementary information [file ELSC-21-258-s001.pdf]

Engineering in Life Sciences

Supporting Information

***Pseudomonas taiwanensis* biofilms for continuous conversion of cyclohexanone in drip flow and rotating bed reactors**

Ingeborg Heuschkel[1], Selina Hanisch[1,2], Daniel C. Volke[3], Erik Löfgren[4], Anna Hoschek[1], Pablo I. Nikel[3], Rohan Karande[1], Katja Bühler[1,2]

[1] Department of Solar Materials

Helmholtz-Centre for Environmental Research—UFZ GmbH

Leipzig, Germany

[2] ZINT - Zentrum für integrierte Naturstofftechnik

TU Dresden

Dresden, Germany

[3] The Novo Nordisk Foundation Center for Biosustainability

Lyngby, Denmark

[4] SpinChem AB

Umeå, Sweden

*Correspondence:

Rohan Karande

E-mail: rohan.karande@ufz.de

1 Bacterial strains and plasmids

All bacterial strains and plasmids used are listed in Table S1. Plasmids were constructed following standard procedures, DNA manipulation, as well as agarose gel electrophoresis, were performed as described by Sambrook and Russell (2001) and E. coli DH5α was used for cloning. Detailed information on primers used are provided in Table S2. Primers were obtained from Eurofins Genomics (Ebersberg, Germany). The gene encoding for BVMO was amplified with the primers BVMO for and BVMO rev from pCom10_capro at an annealing temperature of 64 °C and an elongation of 5 s. The amplified BVMO, as well as DNA fragments for the terminator, were cloned into the empty cloning vector pRSF_Ptrc1O:Term via the restriction sites SpeI and XbaI using Gibson assembly.

For the amplification of the *DGC* gene the following primes were used (Table S3). Both the PCR product and vector pSEVA6311 (Martínez-García et al., 2020) were digested with SacI and BamHI, and the fragments were column-purified and ligated. E. coli DH5α was transformed with the resulting ligation mixture and selected on agarose plates containing gentamycin. The correctness of the resulting plasmid (pS6311::DGC-244) was verified by sequencing.

Electrocompetent Pseudomonas cells were prepared according to Choi and Schweizer (2006) and transformation performed by electroporation (2500 V, Eppendorf Eporator, Hamburg, Germany).

Table S1: Bacterial strains and plasmids used for cloning

Strain/Plasmid	Description	Reference
E. coli DH5α	F$^-$ φ80*lac*ZΔM15 Δ(*lacX*ZYA-*arg*F) U169 *rec*A1 *end*A1 *hsd*R17(rK$^-$, mK$^+$) *pho*A *sup*E44 λ^- *thi*-1 *gyr*A96 *rel*A1	(Hanahan et al., 1983)
Pseudomonas sp. VLB120	Wild-type *Pseudomonas*	(Panke et al., 1998)
pRSF_Ptrc1O:Term	pPMQAK1 based, RSF ori, P_{rnpB}:lac*I*, P_{trc1O}:*Term*, empty cloning vector	(Hoschek et al., 2017)
pCom10_capro	pCom10 derivative, with genes encoding for Cyp P450 monooxygenase (CHX), ferredoxin reductase (FdR), ferredoxin (Fd), cyclohexanone monooxygenase (BVMO) and cyclohexanol dehydrogenase (CDH) from *Acidovorax* CHX100	(Karande et al., 2017)
pSB1AC3_Ptrc1O:GFP mut3B	pMB1, biobrick #BBa_B0015 terminator sequence	(Huang et al., 2010)
pSEVA6311	pBBR1 ori, P_{chnR}-P_{chnB} promoter, Gm resistance	(Martínez-García et al., 2020)
pRSF_Ptrc1O:BVMO	pPmQAK1, RSF ori, P_{rnpB}:lac*I*, P_{trc1O}:BVMO	This study
pS6311::DGC-244	pSEVA collection: pBBR1 ori, Gm resistance, P_{chnR}-P_{chnB} promoter (Benedetti et al., 2016), diguanylate cyclase	This study

Table S2: Primers used for the construction of pRSF_Ptrc1O:BVMO

Primer funtion	Sequence
BVMO fwd	TGAGCGGATAACAATTTCACACATACTAGAG**TAGTGGAGGT**TACTAG*ATGAAAAAAACCCAACATCTGG*
BVMO rev	CTTTCGTTTTATTTGATGCCTGGTACTA**TTTTTCGAACTGCGGGTGGCTCCA**AGCGCT*CTGGAATACGAAACCCTCG*
Term fwd	GGGAGGTATTGGACCGCATTGAACTCTAGTA*TATAAACGCAGAAAGGCCC*
Term rev	ACGAGCCGGATGATTAATTGTCAATCTAGAG*CCAGGCATCAAATAAAACG*

overlap to vector, scar, **RBS***, *binding region*

Table S3: Primers used for the construction of pS6311::DGC-244

Primer funtion	Sequence
DGC fwd	AAAGAGCTCTTA**GGAGGAAAAACAT**ATGAAAATCTCAGGCGCCCGGAC
DGC rev	AAAGGATCCTCA AGC GCT CCT GCG CTTG

restriction site, **RBS***

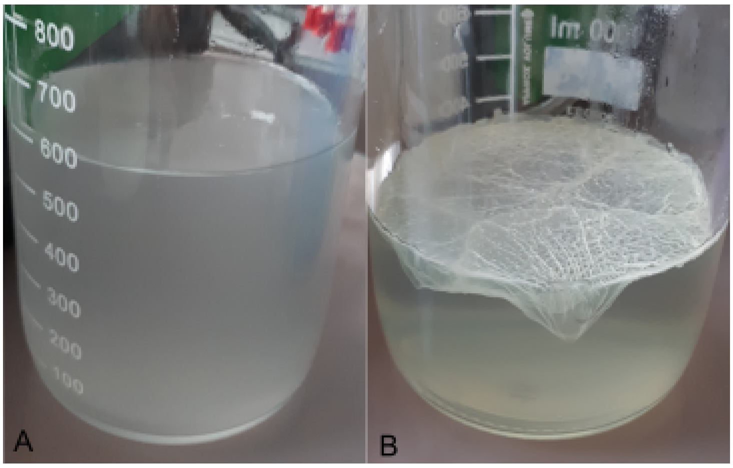

Fig. S1: Suspended growth of Ps_BVMO (A) and biofilm formation on the medium surface of Ps_BVMO_DGC (B) in the waste bottles of the DFRs.

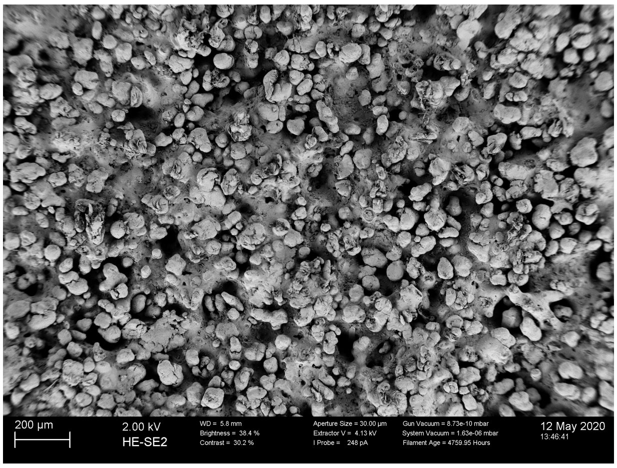

Fig. S2: SEM image of the nylon coupon surface

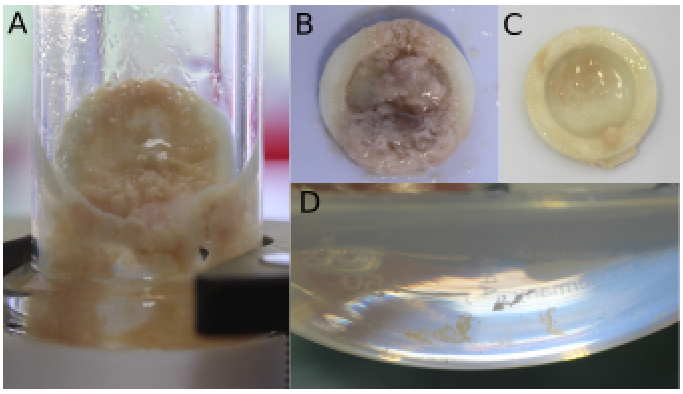

Fig. S3: Biofilm on the coupon in the DFR (A). The coupon was removed from the DFR (B), shaken for 60 min (150 rpm) in a shake flask (M9* medium) and removed afterwards (C). Flocks of biomass separated from the coupon (D).

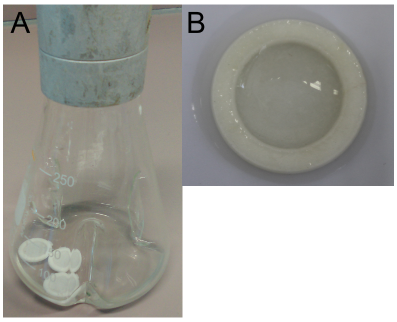

Fig. S4: Biomass growth on the coupons in static conditions. Coupons were added to a shake flask containing M9* medium (A), medium was exchanged after 24 h and the coupons removed after 9 days of cultivation (B).

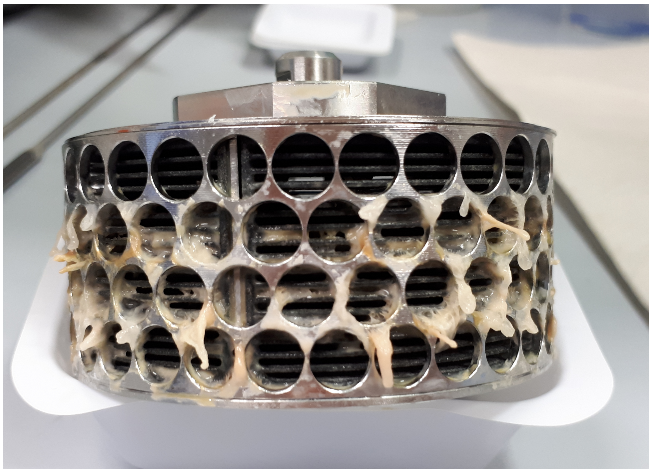

Fig. S5: Biofilm spikes were formed at 400 rpm (Ps_BVMO_DGC) increasing the surface for biofilm growth by inhabiting space outside the RBBR.

Supporting References

J. Sambrook, D. Russell, *Cold Spring Habour Laboratory Press, New York* **2001**

Choi, K. H., & Schweizer, H. P., *Nature protocols* **2006**, 1, 1

D. Hanahan, *J. Mol. Biol.* **1983**, 166, 557

Panke, S., Witholt, B., Schmid, A., Wubbolts, M. G. , *Appl. and Environ. Microbio.* **1998**, 6, 64

A. Hoschek, B. Bühler, A. Schmid, *Angew. Chem.*, Int. Ed. **2017**, 56, 15146

R. Karande, D. Salamanca, A. Schmid, K. Buehler, *Biotechnol. Bioeng.* **2017**, 115, 312

H. H. Huang, D. Camsund, P. Lindblad, T. Heidorn, *Nucleic Acids Res.* **2010**, 38, 2577

Martínez-García, E., Goñi-Moreno, A., Bartley, B., McLaughlin, J., Sánchez-Sampedro, L., Del Pozo, H. P., et al., *Nucleic Acids Res.* **2020**, 48, 3395

Benedetti, I., de Lorenzo, V., Nikel, P. I., *Metab. Eng.* **2016**, 33
